# Off-Screen Sound Separation Based on Audio-visual Pre-training Using Binaural Audio

**DOI:** 10.3390/s23094540

**Published:** 2023-05-07

**Authors:** Masaki Yoshida, Ren Togo, Takahiro Ogawa, Miki Haseyama

**Affiliations:** 1 Graduate School of Information Science and Technology, Hokkaido University, N-14, W-9, Kita-ku, Sapporo 060-0814, Hokkaido, Japan; 2 Faculty of Information Science and Technology, Hokkaido University, N-14, W-9, Kita-ku, Sapporo 060-0814, Hokkaido, Japan

**Keywords:** audio-visual systems, off-screen sound, sound source separation, pre-training, binaural audio

## Abstract

This study proposes a novel off-screen sound separation method based on audio-visual pre-training. In the field of audio-visual analysis, researchers have leveraged visual information for audio manipulation tasks, such as sound source separation. Although such audio manipulation tasks are based on correspondences between audio and video, these correspondences are not always established. Specifically, sounds coming from outside a screen have no audio-visual correspondences and thus interfere with conventional audio-visual learning. The proposed method separates such off-screen sounds based on their arrival directions using binaural audio, which provides us with three-dimensional sensation. Furthermore, we propose a new pre-training method that can consider the off-screen space and use the obtained representation to improve off-screen sound separation. Consequently, the proposed method can separate off-screen sounds irrespective of the direction from which they arrive. We conducted our evaluation using generated video data to circumvent the problem of difficulty in collecting ground truth for off-screen sounds. We confirmed the effectiveness of our methods through off-screen sound detection and separation tasks.

## 1. Introduction

Humans can easily recognize and separate each sound, even in a noisy environment, which is known as the “cocktail party effect” [1]. This effect is significantly assisted by visual information [2]. For example, a speaker’s appearance enables us to assume the speaker’s voice, the speaker’s lip or body motion indicates the timing of their speech, and the speaker’s position indicates the direction from which their voice is coming when using both ears. We can recognize sound in these ways because vision and sound have strong correlation and correspondences in the semantic, temporal, and spatial dimensions. Based on this correlation, humans can unconsciously combine visual and auditory perception to obtain rich sensory information [3,4]. Focusing on this correlation and the human recognition of sound, researchers have been exploring audio-visual (A-V) learning, such as speech separation [5,6], A-V source separation [7,8,9,10], and A-V self-supervised learning [11,12,13]. A-V learning has overcome the limitation of recognition tasks with a single modality by using multiple modalities. Most of the recently proposed methods are built on the basis of deep learning. This research field is rapidly growing, and many methods for advanced video recognition have been proposed [14]. This suggests that the relationship between vision and sound can be represented and controlled by machines. This trend raises users’ expectations for machine-learning-based audio and video manipulation.

With the rapid development of multimedia content-sharing services [15], there is a growing demand for users to have complete control over audio and video. A-V learning has great potential for various applications such as the separation of sounds from videos [7], automatic sound generation from videos [16], and the conversion of mono audio into binaural audio [17]. It is expected that the advancement of A-V learning will make it possible to freely manipulate the relationship between videos and sounds on a computer. The free manipulation of audio and video can not only provide a new experience to video viewers but also aid video production.

**Figure 1:**
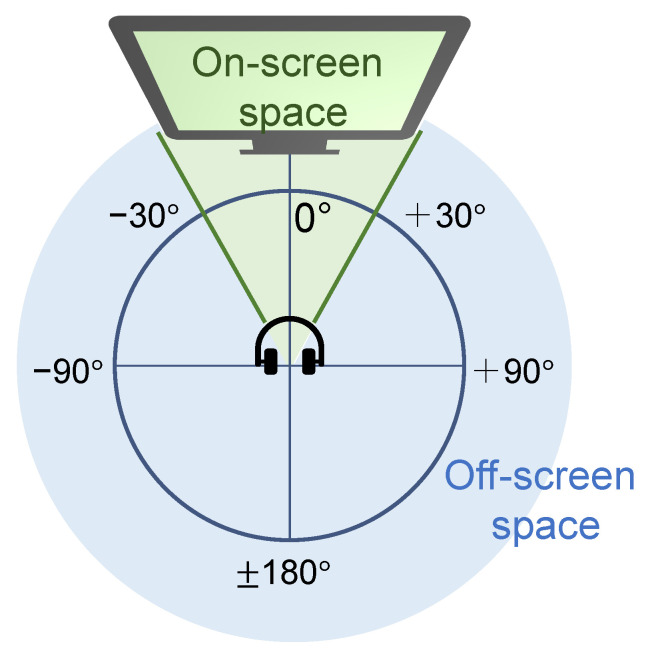
On-screen and off-screen space set in this study. We call sounds coming from the blue area OSSs.

Although A-V learning has considerable potential in its correlation between audio and video, the assumption does not always hold in real-world videos. Specifically, off-screen sounds (OSSs), whose sources are invisible in videos, have no correspondence between audio and video. Note that we define on-screen, as shown in Figure 1, based on the space of sounds. The space from a user to a screen is defined as the on-screen space, and sound coming from the other space is defined as the OSS. This definition clarifies the off-screen space and allows the consideration of the arrival directions of OSSs. OSSs are not related to the contents on the screen; namely, the information about OSSs is included in the audio stream but not in the visual stream. OSSs cause performance degradation in A-V learning due to the degradation of the A-V correlation. For video viewers, OSSs are typically undesired sounds and prevent viewers from concentrating on the content on the screen. Hence, detecting and separating OSSs is an essential technique for freely controlling audio and video in A-V learning.

A common approach to A-V source separation [7,8] based on the mix-and-separate strategy [7] is to use visual information as guidance for the separation. Concretely, this approach attempts to separate a sound mixture into each sound corresponding to given visual frames, where all sound sources are assumed to be in the videos. However, if OSSs are mixed in the training data, these models cannot use the visual images of OSSs and fail to separate them. To tackle this problem, several OSS separation methods have been proposed. Owens et al. [12] explored an A-V self-supervised learning method that learns an A-V representation based on the A-V temporal correspondence and demonstrated OSS separation using the learned representation. Yang et al. [13] proposed another A-V self-supervised learning method using binaural audio that allows listeners to identify the arrival directions of sounds. Although these studies considered temporal or spatial A-V correspondence, they did not consider off-screen space or sounds. Tzinis et al. [9,10] proposed an A-V source separation method based on mono audio that separates each sound and classifies whether each sound is inside or outside the screen. Instead of identifying OSSs after separation, we used spatial sounds and more directly separate OSSs by focusing on their characteristics. Briefly, the following issues must be addressed in order to separate OSSs. Conventional separation methods assume that all sound sources are present in the video frame. Therefore, the data are insufficient to account for the presence of off-screen sounds. Spatial audio-visual correspondence, which is limited to the space within the screen, cannot handle off-screen sound. It is difficult to achieve the accurate separation of off-screen sound by solely relying on A-V correlation.

In this study, we developed a novel OSS separation method with binaural audio based on A-V pre-training. We expected that the A-V pre-trained representation that considers OSS characteristics would enable us to directly separate OSSs. Our key insights are as follows: (i) The A-V spatial correlation enabled us to directly separate OSSs because OSSs come from outside the screen. (ii) The proposed A-V pre-training method considering OSS characteristics is suitable for OSS separation. Based on the above key insights, we introduce a new A-V pre-trained representation into the OSS separation process based on the arrival directions of sounds. In the proposed method, we artificially generate OSSs and use them for the pre-training and OSS separation. We solved the problem of the lack of data for OSSs by generating the data ourselves. This data generation enabled the learning and evaluation of OSS separation. The generation of OSSs enabled us to obtain an A-V representation based on OSS characteristics in our pre-training method. By considering both on-screen and off-screen space through pre-training, it was possible to obtain an effective audio-visual feature representation for separating OSSs. In the OSS separation training, the generated OSSs in various directions were available as the ground truth of separation based on the arrival directions of the sounds. Thus, the proposed method was designed to learn the knowledge of OSSs to separate OSSs arriving from any direction. By incorporating both A-V correspondence and arrival direction considerations through this separation learning method, high-precision separation was achieved.

One simple application of our method is eliminating unwanted OSSs so that the viewer can focus on the on-screen content. Binaural audio is expected to be used with VR/AR technology, where three-dimensional video and audio are required due to the use of head-mounted displays and AR glasses. Therefore, our method can be applied in the future, for example, to remove noise outside the visible range of the AR glasses. Furthermore, there is research on the use of binaural audio for humanoid robots [18], and our method is expected to be applied to A-V methods for robots used for, e.g., robotic navigation.

## 2. Related Works

### 2.1. Audio-Visual Source Separation

A-V source separation uses visual information to separate each sound from a sound mixture. Recent studies have applied self-supervised learning to A-V source separation rather than using annotated audio and video data. First, Zhao et al. [7] proposed the mix-and-separate framework, which generates a sound mixture from soundtracks and predicts each sound using its corresponding visual frames. This framework can extract sound source information from visual frames and use it as supervision for the separation. This mix-and-separate framework has been commonly used in subsequent studies [8,19]. To effectively use the sound source information in visual frames, Gao et al. [8] detected and cropped object areas in visual images and used the cropped images as supervision. In addition, Tian et al. [19] excluded silent objects from detected objects because silent object images create confusion during source separation. These approaches are intended to accurately use the sound source information in visual frames. Contrary to these approaches, we focused on sound objects not shown in videos. We also adopted a pre-training approach based on the mix-and-separate framework and performed OSS separation by creating OSSs from unlabeled data.

### 2.2. Pre-Training in Videos

In A-V research, pre-training is used to learn the relationship between audio and video, and the obtained representations and models can be used in various downstream tasks. Particularly, self-supervised learning does not require annotation and has been reported to be effective for various A-V tasks [11,12,13]. A-V self-supervised learning aims to obtain a useful A-V feature for various types of A-V learning. A useful A-V feature has learned correlations between audio and video. Conventional studies have focused on semantic [11], temporal [12], and spatial [13] correspondences. In these studies, the A-V model learns whether the input audio semantically, temporally, or spatially matches the video. Among these correspondences, spatial information is considered to play an essential role in OSS separation because sounds coming from off-screen spatial locations are OSSs. Yang et al. [13] focused on spatial information by assigning to the video the task of determining whether the audio is flipped left or right; the off-screen space was not considered. Accordingly, a pre-training method that considers the off-screen space is required. Regarding conventional studies that used OSSs as supervision, Owens et al. [20] indicated that ambient sounds are available for scene recognition, as the sound of wind corresponds to an outdoor scene. It may be true that OSSs can be used as supervision for scene recognition; however, OSSs may weaken the A-V correlations. Thus, we adopted a strategy to perform pre-training for separate OSSs and predict the inclusion of OSSs to understand the A-V correlation.

### 2.3. Audio-Visual Learning Using Binaural Audio

In our study, we used binaural audio, which is two-channel audio acquired by a microphone built into a dummy head. The dual duplex means that humans can localize sound locations primarily by using the time difference (interaural time difference) and the level difference (interaural level difference) of sounds reaching the two ears [21,22]. Therefore, binaural audio contains spatial information of sound sources, which is critical for sound source separation. Binaural audio has been investigated in the fields of auditory neurobiology [23] and speech signal processing [24] and has recently attracted attention in the field of A-V learning with deep learning. Researchers have used binaural audio for creating video datasets [13,17,25] sound localization [26], binaural audio generation [27], and object prediction [28]. We also used binaural audio to determine the arrival directions of sounds and separate OSSs.

## 3. Proposed Method

**Figure 2:**
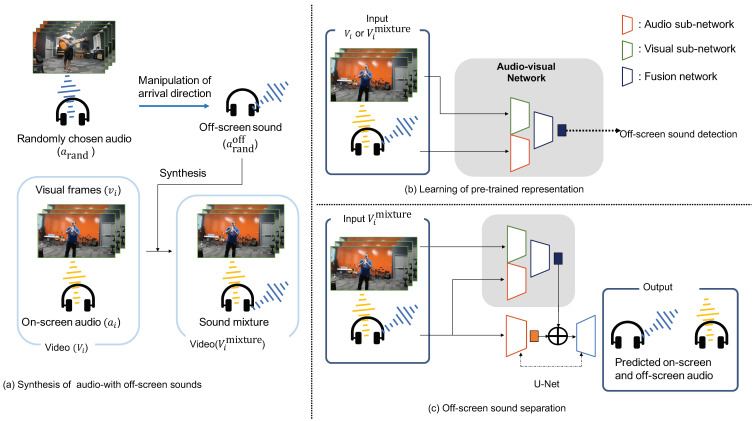
Overview of the proposed method: (**a**) artificial synthesis of audio with OSSs, (**b**) OSS detection to learn a useful pre-trained representation, and (**c**) separation of OSSs based on the pre-rained representation.

Figure 2 provides an overview of the proposed method. The proposed method consists of the following three phases: synthesizing OSSs, obtaining a pre-trained representation, and OSS separation using the learned representation. A summary of each phase is provided below.

**Phase (a)**  We generated data for use in subsequent phases. Specifically, we generated labels for Phase (b) and the ground truth for Phase (c). ( Section 3.1)
**Phase (b)**  We conducted pre-training to obtain an effective audio-visual representation for OSS separation. The task was the detection of OSSs. (Section 3.2)
**Phase (c)**  We trained a model for separating OSSs, which is the primary objective of our method. (Section 3.3)

The details of each phase are presented in the following subsections.

### 3.1. Synthesizing Off-Screen Sound

Humans have an effective visual field that is strongly related to concentration. The effective visual field is significantly influenced by stimuli in the surrounding environment, and the effective horizontal field of view is generally approximately $30^{\circ }$ in both directions [29]. According to this mechanism, we defined the off-screen space, shown in Figure 1. The relationship between the arrival directions of sounds and visual input is also an important factor in A-V learning.

To use the arrival directions of sounds, it is necessary to prepare audio data for various arrival directions. However, there was no audio dataset labeled with the arrival directions of sounds. In addition, we could not obtain the ground truth of OSSs and on-screen sounds from the audio datasets that already contain OSSs. Hence, we first prepared the data $D^{\mathrm{on}}$ without OSSs and artificially manipulated the arrival directions of sounds in $D^{\mathrm{on}}$ to prepare the OSS data. We prepared on-screen videos (without OSSs) $D^{\mathrm{on}}={\left\{V_{i}\right\}}_{i=1}^{N}={\left\{\left(v_{i},a_{i}\right)\right\}}_{i=1}^{N}$, where $V_{i}$ represents a video, including visual frames and audio tracks; $v_{i}$ represents visual frames; and $a_{i}$ represents an audio track. Furthermore, *N* indicates the number of videos. Based on this definition, we created the synthesized dataset for OSS separation. First, we randomly chose track $a_{\mathrm{rand}}$ from $D^{\mathrm{on}}$ and manipulated it so that the arrival direction became off-screen as follows:

(1) $$a_{\mathrm{rand}}^{\mathrm{off}}=f_{\varphi ,\vartheta }\left(a_{\mathrm{rand}}\right),$$

where $a_{\mathrm{rand}}^{\mathrm{off}}$ is an OSS generated from $a_{\mathrm{rand}}$; $f_{\varphi ,\vartheta }\left(\cdot \right)$ represents the manipulation of the arrival direction; and φ and ϑ denote the arrival directions of the azimuth and zenith angles, respectively. We used this track $a_{\mathrm{rand}}^{\mathrm{off}}$ as OSS and add it to $v_{i}$:

(2) $$\begin{array}{cc} V_{i}^{\mathrm{mixture}} & =V_{i}+a_{\mathrm{rand}}^{\mathrm{off}}\\ \; & =(v_{i},\;a_{i}+f_{\varphi ,\vartheta }\left(a_{\mathrm{rand}}\right)), \end{array}$$

where $V_{i}^{\mathrm{mixture}}$ represents the *i*th video with a sound mixture that includes OSSs. This synthesis is visualized in Figure 2a. We applied this synthesis to videos $V_{i}\;\left(i=1,2,\cdots ,N\right)$ and defined these synthesized videos as $D^{\mathrm{mixture}}$.

We used PseudoBinaural [27] to manipulate the arrival direction of audio tracks. PseudoBinaural adopts spherical harmonic decomposition [30] and head-related impulse response (HRIR) [31] open-sourced data [32] and can convert mono audio into binaural audio in any direction. PseudoBinaural leverages the binaural rendering technique that combines ambisonics with HRIR [33]. This approach more accurately renders binaural audio than the open-source HRIR [32], which is recorded in a free field and cannot recover the precise binaural effect due to the reverberation in normal scenes. For our proposed method, we converted prepared binaural audio $a_{i}$ into mono audio and then converted the mono audio into OSSs using PseudoBinaural. Because the angle of view and the range of a video image vary depending on the camera, we evenly set the range of the video image, as shown in Figure 1. For more details, we set φ to be in the range of $\pm \left[30^{\circ },180^{\circ }\right]$ and ϑ to be $0^{\circ }$.

Our generation of OSSs enabled us to prepare a large amount of audio with OSSs coming from different directions by randomly setting φ in the range of $\pm \left[30^{\circ },180^{\circ }\right]$. Furthermore, we could automatically obtain labels indicating the contamination of OSSs and used these labels in our representation learning method. We also used $a_{i}$ and $a_{\mathrm{rand}}^{\mathrm{off}}$ as the ground truth of on-screen and off-screen tracks in the OSS separation process. In addition, although the number of audio and video pairs in $D^{\mathrm{on}}$ are limited to *N*, we could generate many videos and OSS pairs by changing the $a_{\mathrm{rand}}$ and φ. This resulted in a data augmentation effect.

### 3.2. Obtaining a Pre-Trained Audio-Visual Representation

**Figure 3:**
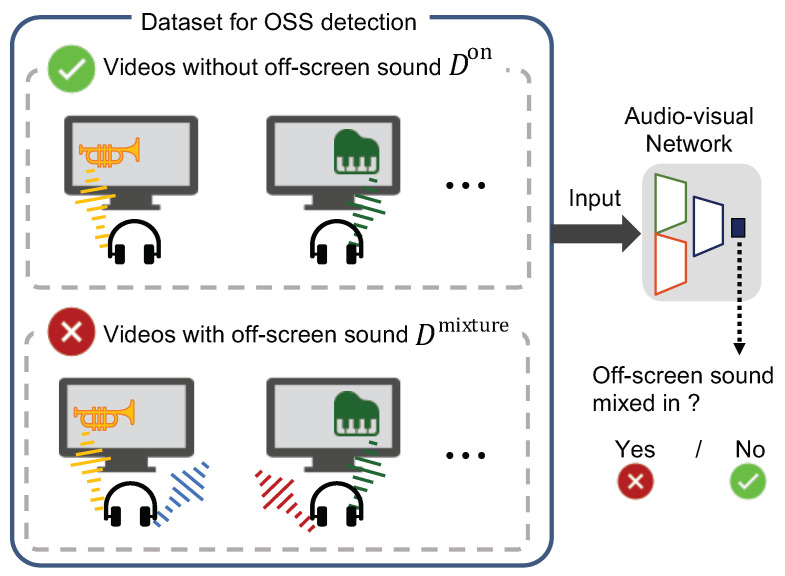
Our pre-training method. The A-V network learns to detect OSSs. OSSs are mixed when the arrival directions of sounds do not correspond to the audio and video.

The most significant characteristic of OSSs is that OSSs come from off-screen space. Motivated by this, we developed a new sophisticated A-V representation learning method based on OSS detection. OSS detection is visualized in Figure 3. The OSS detection task should focus on A-V spatial correspondences and teach the network the directions of OSSs. Then, an A-V representation is obtained based on spatial correspondences. The obtained representation is useful for downstream A-V tasks such as OSS separation. We first constructed an A-V network for OSS detection, as shown in Figure 2b. The network consisted of the following three types of networks [13]: an audio subnetwork, a visual subnetwork, and a fusion network. We used stacked residual blocks with S & E [34] as our base model for the audio subnetwork and used ResNet-18 [35] as our base model for the visual subnetwork. The fusion network comprises a convolutional layer, a global average pooling layer, and a fully connected layer. As an input to the audio subnetwork, the left and right channels are transferred into their spectrograms in the time-frequency domain using the Short-Time Fourier Transformation (STFT). The left and right spectrograms have real and imaginary channels, and we stacked them to make a four-channel input spectrogram. We then input the spectrogram and visual frames into the audio subnetwork and the visual subnetwork, separately, to obtain the features from both subnetworks. The audio feature has dimensions [*C*, *T*, *F*], where *C*, *T*, and *F* denote the channel, time, and frequency dimensions, respectively. The visual feature has dimensions [*C*, *T*, *W*, *H*], which are the channel, time, width, and height dimensions, respectively. We concatenated the audio and visual features along their time dimensions *T*. Before this concatenation, we flattened the visual feature along the *W* and *H* dimensions without pooling and tiled the visual feature to fit the dimensions of the audio feature. The flattening without pooling was necessary to use the positions of sound sources in the visual subnetwork because the pooling of height and width dimensions reduces the positional information in the visual feature [13]. Finally, the concatenated A-V feature was processed by the fusion network to predict the probability that the input mixture contained OSSs.

We used both the $D^{\mathrm{on}}$ and $D^{\mathrm{mixture}}$ datasets as the negative and positive samples for OSS detection, respectively. Specifically, while training the A-V network, let $D=D^{\mathrm{on}}\cup D^{\mathrm{mixture}}\;={\left\{{\left(v,a,y\right)}_{i}\right\}}_{i=1}^{N}$ be our video dataset, where *y* indicates whether OSSs are contaminated in audio *a*. Note that the pseudo label *y* can automatically be obtained using the characteristics of the audio and video correspondence. For the network training, the A-V network g(v,a) maximizes a classification cross-entropy objective given by the log-likelihood as follows:

(3) $$V=\sum_{(v,a,y)\in D}y\log g(v,a)+\left(1-y\right)\log\left(1-g(v,a)\right).$$

Generating $D^{\mathrm{mixture}}$ from $D^{\mathrm{on}}$ in Section 3.1 automatically produces positive and negative examples for the OSS contamination, and we can perform this pre-training.

In $D^{\mathrm{on}}$, the sound source position in the video corresponds to the arrival direction of sounds. On the other hand, in $D^{\mathrm{mixture}}$, OSSs reduce the spatial correspondence between the video and audio because OSSs are not visible in the video. By distinguishing these differences, the A-V network learns the spatial correspondences to detect OSSs. The spatial correspondences are critical information not only for OSS separation but also for other A-V tasks using binaural audio. Thus, the A-V network can be introduced into various A-V tasks and improves their performance.

### 3.3. OSS Separation

General audio source separation aims to obtain an estimation of each sound source from the observed sound mixture. However, our goal was to separate the sound mixture into OSSs and on-screen sounds. Furthermore, we separated the OSSs based on their arrival directions. To this end, we introduced PseudoBinaural into the OSS separation training.

The separation model is outlined in Figure 2c. We constructed the separation framework on the basis of the mix-and-separate strategy [7] adapted for stereo audio [17]. Specifically, we used the A-V network in Section 3.2 and the same U-Net [36] as in the previous methods [17] to predict the complex masks and separate OSSs. The original mix-and-separate strategy requires each visual frame to be accompanied by its audio for separation. However, the sources of the visual frames of OSSs are unknown. Hence, we only provided the model with on-screen visual frames in visual processing. For audio input, we provided the model with the stacked left and right channel spectrograms obtained by STFT on the sound mixture generated from the on-screen audio and OSS in Section 3.1. Because we had both on-screen and off-screen audio available, we used them as the ground truth for OSS separation. The separation model took the above inputs and processed them as follows: The innermost layer of the U-Net was concatenated with the feature before the global average pooling layer in the fusion network. The decoder of the U-Net predicted complex masks for on-screen and off-screen audio, separately. We multiplied the masks and input spectrograms to obtain the spectrograms of the on-screen and off-screen audio. By applying inverse short-time Fourier transformation (ISTFT) on the obtained spectrograms, we finally obtained their waveforms. Because we could use the ground truth spectrograms in our framework, we followed previous studies [7,8] and minimized the per-pixel L1 loss [37] between the ground truth and the predicted spectrograms as follows:

(4) $$L=∥S_{\mathrm{on}}-{\tilde{S}}_{\mathrm{on}}{∥}_{1}+{∥S_{\mathrm{off}}-{\tilde{S}}_{\mathrm{off}}∥}_{1},$$

where $S_{\mathrm{on}}$ and $S_{\mathrm{off}}$ are, respectively, the spectrograms of the on-screen sounds and OSSs; and ${\tilde{S}}_{\mathrm{on}}$ and ${\tilde{S}}_{\mathrm{off}}$ are our model’s predictions of them. By creating OSSs coming from the off-screen space and using them as the ground truth of separation, the separation model could learn to separate sounds coming from the off-screen space. Furthermore, the introduction of our pre-trained representation makes the separation model use the knowledge of the off-screen space. In this way, our method can achieve successful OSS separation.

## 4. Experiments

We conducted the following two types of experiments: OSS detection and separation. For OSS detection, we evaluated whether our representation could learn the A-V correlation. Furthermore, the accuracy of our method was evaluated in terms of OSS separation.

Before describing each experiment, we explain the dataset used in both experiments. We used the FAIR-Play dataset [17] comprising 1871 videos of 10 s clips recorded in a music room. The videos are paired with binaural audio tracks that are only on-screen sounds. In terms of content, each video has approximately 1–3 sound sources, and there are approximately 10 types of sound sources. We applied the following processing to video and audio, separately. We used 5.11 s videos clipped at a random start time from the 10 s videos. The video frames were sampled at 10 Hz and resized to 256 × 512 pixels. For data augmentation, we used random color and contrast shifts to augment the data. The audio input was sampled at 16 kHz and normalized to have a constant root mean square amplitude. We processed the audio with STFT (Hann window, window size = 400, number of frequency bins = 512, and hop size = 160). We split the data into training, validation, and test sets of 1497, 187, and 187 clips, respectively. Because the FAIR-Play dataset consists of only on-screen sounds, we generated a sound mixture with OSSs in each set and used them in the following experiments. To avoid varying accuracy depending on the included OSSs, we used fixed video and OSS pairs in the validation and test sets.

### 4.1. OSS Detection

We first demonstrate the results of the OSS detection task to confirm the extent to which the proposed pre-trained representation has learned the A-V correlation.

#### 4.1.1. Settings

We input audio samples with or without OSSs into the A-V network trained in Section 3.2 and verified that it could correctly detect OSSs. We input the audio sample with OSSs as a positive sample. Our method detects OSSs by focusing on visual information and the arrival directions of sounds. We performed the following to confirm the reasonableness of our method. To assess the need for the arrival directions of sounds, we used the representation acquired by learning with monaural audio (mono learning), where the sound directions were unavailable. Additionally, to assess the need for visual information, we removed the visual subnetwork from our network and used only audio information to detect OSSs (no-visual).

We evaluated OSS detection performance using audio in the following six different cases:

(1) Audio without any OSS.
(2) Audio converted to mono from the entire audio described in (1).
(3) Audio with OSS.
(4) Audio with OSS. The OSS in this case was created by manipulating the arrival direction of the on-screen sound. Here, because the semantic information about on-screen sounds and OSSs was the same, only the spatial information about the sound was used for detection.
(5) Audio that did not visually appear on the screen but arrived from the on-screen direction.
(6) Audio converted to mono from the entire audio described in (3).

Cases (1) and (2) contained no OSS. For (3) and (4), off-screen sound was included. Additionally, (5) and (6) included audio that was not defined as OSS, but the sound source did not appear on the screen. These were classified as pseudo-OSS and considered negative.

To evaluate the overall detection accuracy, we used the area under the ROC curve (AUC). Additionally, we used the true-negative rate (TNR) for cases (3) and (4), and the true-positive rate (TPR) for the other cases.

#### 4.1.2. Experimental Results

**Figure 4:**
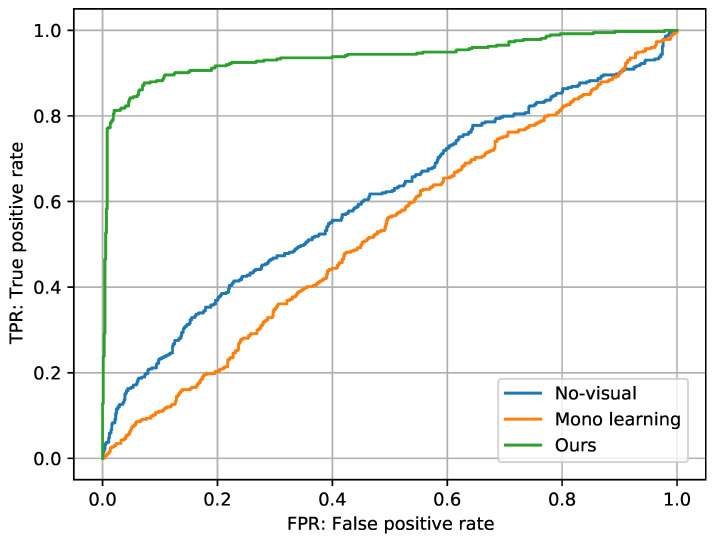
The ROC curves of the methods.

We present the detection results in Table 1. Additionally, we show the ROC curves in Figure 4. Our method outperformed the other methods in terms of AUC. This result indicates that spatial audio information and visual information are necessary to detect OSSs. The results for the cases were as follows: We can see that our method could detect OSSs with high accuracy in cases (3) and (4) and correctly identified the negative samples in cases (1), (2), (5), and (6), including pseudo-OSS. In addition, our representation could detect OSS even when on-screen sounds and OSSs were the same in case (4). These results indicate that our representation learned the A-V correlation and detected OSSs on the basis of the arrival directions of sounds. Additionally, the results that the representation by mono learning and without visuals had high true-positive rates but low true-negative rates, indicating that these representations could not accurately detect OSSs. The result of the no-visual method could have been due to errors in identifying the front and back directions. Front–back localization is more difficult than left–right localization because the interaural time and level differences, the most important localization cues, are unavailable. Although humans can discriminate front–back using spectral cues that depend on head shadows and ear shape [38], they can also make mistakes [39]. Furthermore, there is no restriction on the source type or number of sources in the input audio: it is necessary to find spectral cues that indicate off-screen directions in the spectrogram of the mixture. On the other hand, in the proposed method, the visual information provides the type and number of sound sources on the screen, so the OSS contamination can be identified not only from the spectral cue but also from the correspondence between the visual and auditory information. These results suggest that it is important to combine visual and auditory spatial information.

### 4.2. OSS Separation

To evaluate the effectiveness of the proposed method, we conducted the OSS separation task.

#### 4.2.1. Settings

We input audio samples with OSSs into our separation model trained as described in Section 3.3 and verified that it could correctly separate OSSs. To also examine the variation in separation accuracy depending on the arrival directions of OSSs, we set the arrival directions of OSSs from $30^{\circ }$ to $180^{\circ }$ by $30^{\circ }$. We compared our method with conventional A-V source separation methods [7,8] to evaluate the overall separation performance of the proposed separation method. These comparison methods (CMs) are detailed below.

**CM1.**  This method [7] uses the mix-and-separate framework and visual information for separation.
**CM2.**  This method [8] first detects and obtains candidates of sound sources and uses the detected results for separation.

Moreover, we replaced our pre-trained representation with other features to evaluate the effectiveness of our pre-training method.

**CM3.**  This method is solely based on semantic representation learning [11].
**CM4.**  This method is solely based on spatial representation learning [13].
**AB1.**  For the ablation studies, this method uses a randomly initialized feature without pre-training.
**AB2.**  For ablation studies, this method uses a feature obtained from mono learning as described in Section 4.1.
**AB3.**  For ablation studies, this method removes the visual subnetwork and uses only audio information. This method uses the feature obtained from the no-visual learning in Section 4.1.

All methods used binaural audio as the input. If the original CMs took monaural audio as the input, we doubled the input channels in the first layer of the model to support binaural audio. Supplementally, we compared our method with beamforming methods, which are classic source separation methods based on the arrival direction of sound. Note that the experimental conditions for this comparison were different from those of the proposed method, because the environment was hypothetically set up to simulate a recording situation. We used pyroomacoustic [40] to simulate beamforming. The simulation was conducted in the following setup: First, a 10 × 10 m room was prepared in the simulator. We placed two microphones at the center of the room, with the left and right channels 15 cm apart, because the proposed method can only use two channels. We then converted the on-screen sound to mono and placed it 2 m away from the center at 0°. Then, we placed the OSS 2 m away from the center in an off-screen direction similar to that of the proposed method. We computed the beamformer to the angle at which the OSS was placed. We used the commonly used delay-and-sum beamformer (BF1) and the minimum variance distortionless response beamformer (BF2). It was assumed that beamforming for a specific direction was easier in terms of task difficulty than the proposed method of separating all directions from the screen.

As an evaluation metric, we used the L1 distance between the predicted and original spectrograms of OSSs. The L1 distance expresses separation accuracy at the spectrogram level. In addition, we used the scale-invariant SDR (SI-SDR) [41], the scale-invariant SIR (SI-SIR) [41], and the simple signal-to-noise ratio (SNR). These metrics are defined as follows:

(5) $$\mathrm{SISDR}=10\log_{10}\frac{∥s_{\mathrm{target}}{∥}^{2}}{∥e_{\mathrm{interf}}+e_{\mathrm{artif}}{∥}^{2}},$$

(6) $$\mathrm{SISIR}=10\log_{10}\frac{∥s_{\mathrm{target}}{∥}^{2}}{∥e_{\mathrm{interf}}{∥}^{2}},$$

(7) $$\mathrm{SNR}=10\log_{10}\frac{∥s_{\mathrm{target}}{∥}^{2}}{∥s_{\mathrm{target}}-\hat{s}{∥}^{2}},$$

where $s_{\mathrm{target}}$, $e_{\mathrm{interf}}$, and $e_{\mathrm{artif}}$ denote the target sound component, the non-target sound component, and other components in the predicted signal, respectively; $\hat{s}$ is the estimate of $s_{\mathrm{target}}$. Because SI-SDR and SI-SIR were calculated from the reconstructed raw audio, these metrics indicate the separation accuracy at the raw audio level. In order to provide a separation accuracy criterion for the SI-SDR, we additionally report the SI-SDR of the input mixture (MIX). Exceeding this accuracy indicates that the proposed method actually achieved the separation.

#### 4.2.2. Experimental Results

The OSS separation results in terms of L1 distance, SI-SDR, SI-SIR, and SNR are shown in Table 2, Table 3, Table 4 and Table 5, respectively.

As shown in these tables, the proposed method (PM) mostly outperformed the other CMs. For all evaluation metrics, CM1 and CM2 were significantly inferior to the other methods. This is reasonable because these methods do not consider situations in which visual frames are unavailable for separation. In other words, our assumption that OSSs would degrade conventional A-V source separation methods was confirmed, and our separation method was successful in OSS separation. Additionally, neither beamforming method performed accurate separation. Because beamforming is more directional when there are more microphones, it was considered difficult to achieve accurate separation in this experiment using only two microphones. Moreover, comparing PM with CM 3,4 and ABs 1–3, the proposed A-V pre-training method could significantly improve separation performance. Focusing on Table 2, we can see that our representation, which learns the semantic and spatial correspondences, was followed by the representations learning the semantic (CM3) or spatial (CM4) correspondence. This result reveals the effectiveness of our pre-training in OSS detection. Moreover, in all metrics, the separation performance of methods using our separation method described in Section 3.3 (PM, CM3, CM4, and ABs 1–3) increased as the azimuth increased. We evenly set the arrival directions of the OSSs, as shown in Figure 1; however, not all sounds follow edthe setting and on-screen sounds coming from off-screen space would degrade separation performance. Conversely, our separation method attempts to separate OSSs based on their arrival direction, and appropriate settings would improve separation performance. In summary, it was confirmed that our method achieves our goal of separating OSSs based on audio–visual correlation and the arrival direction of sounds.

**Figure 5:**
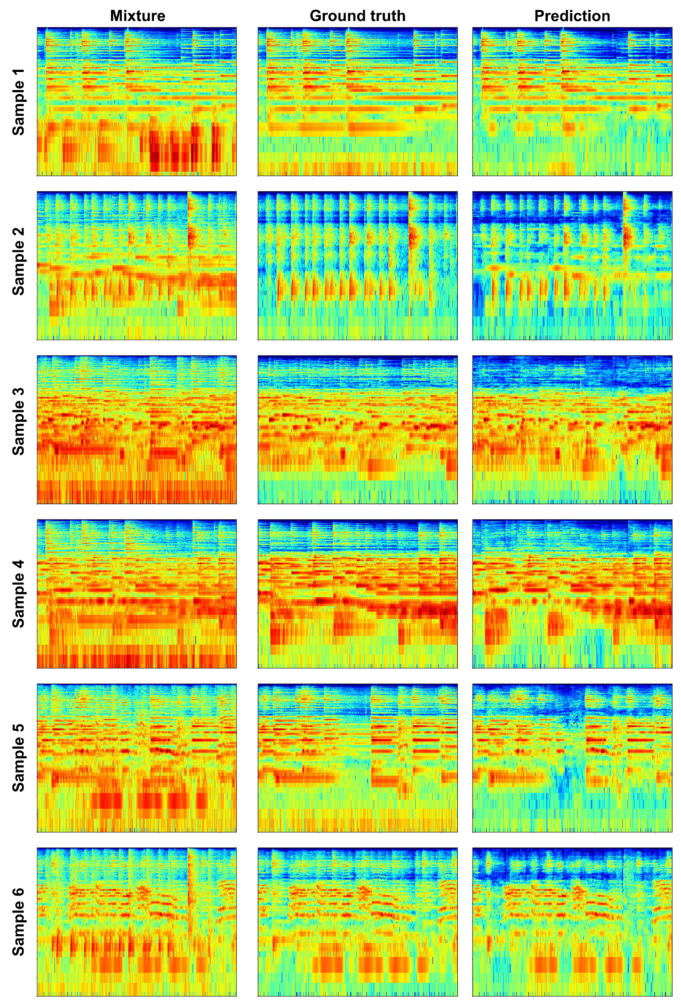
Separation results of the proposed method. We show the log-scale spectrograms obtained from the left channels of the sound mixture, the OSS ground truth, and the OSS predictions. Each spectrogram displays time (0 s to 5.11 s) along the horizontal axis and frequency (0 to 8000 Hz) along the vertical axis. The red indicates more prominent sounds. These spectrograms were obtained using the open-source librosa tool [42]. The accuracy of the separation achieved for each sample was determined by how closely the prediction matched the ground truth.

Furthermore, the spectrogram image samples of the OSS separation results using the PM are shown in Figure 5, which we used to qualitatively evaluate the separation performance. We visualize these spectrogram images as the left-channel results for 5.11 s audio clips. The mixtures were obtained by mixing the ground truth of OSSs with the audio sample paired with the video. Our OSS predictions, although not perfect in every detail, were very close to the ground truth. This indicates that the PM achieved OSS separation with high performance.

## 5. Limitation and Discussion

The limitation of our method is that we need data consisting only of on-screen sounds for training. This requires either labeling the video or capturing the video in an environment where off-screen sounds are not mixed in. To satisfy this requirement, we used the FAIR-Play dataset [17], which was captured in a prepared environment that did not contain OSSs. However, labeling is laborious, and the environments where OSSs do not mixare limited. One way to address this limitation could be to build a pipeline to generate the training data. For example, we can use audio simulators and 3D environment simulators such as SoundSpaces [43], Habitat simulator [44], and Matterport3D [45]. These simulators enable us to generate large and diverse data without the physical limitations of real-world environments. On the other hand, testing on audio with OSSs recorded in real environments is important. Hence, we will consider it as our future work.

## 6. Conclusions

In this paper, we presented a new A-V pre-training method and an OSS separation method through pre-trained representations. We artificially synthesized OSSs, and the synthesized OSSs were essential for our pre-training method and OSS separation method. To obtain a useful pre-trained representation, we made the A-V network detect the contamination of OSSs. OSS detection made the A-V network learn the spatial correspondences between audio and video. We introduced this pre-trained representation into the OSS separation method to improve separation performance. Our separation method used synthesized OSSs set to arrive from off-screen space. We separated OSSs based on their arrival directions by using the synthesized OSSs as the ground truth of separation. Consequently, by learning OSS detection and separation using many directionally manipulated OSSs, the PM obtained knowledge of OSSs and could separate them in any direction from which they arrived. The experimental results show the effectiveness of our pre-training method and OSS separation method.

## Figures and Tables

**Table 1:** OSS detection results. We used AUC to evaluate the overall performance and evaluate details for six cases by using TPR or TNR. Because we detected videos with OSS as positive samples, we used TPR for cases (3) and (4) and TNR for cases (1), (2), (5), and (6).

	w/o OSS			w/ OSS			w/ Pseudo-OSS			AUC
Case	(1)	(2)		(3)	(4)		(5)	(6)		
Ours	0.96	0.95		0.88	0.76		0.96	0.97		0.94
Mono learning	0.25	0.24		0.92	0.79		0.05	0.05		0.53
No visuals	0.11	0.08		0.94	0.89		0.09	0.01		0.60

**Table 2:** OSS separation results: L1 distance. This accuracy indicates the distance between the spectrograms of the input and predicted audio.

Method	$30^{\circ }$	$60^{\circ }$	$90^{\circ }$	$120^{\circ }$	$150^{\circ }$	$180^{\circ }$	Ave
CM1 [7]	1.043	1.045	1.066	1.076	1.086	1.064	1.063
CM2 [8]	0.942	0.929	0.930	0.940	0.946	0.960	0.941
CM3 [11]	0.231	**0.176**	0.149	0.144	0.143	0.136	0.163
CM4 [13]	0.203	0.177	0.160	0.150	0.158	0.139	0.165
AB1	0.286	0.185	0.161	0.156	0.140	0.132	0.177
AB2	0.234	0.178	0.158	0.147	0.140	0.134	0.165
AB3	0.258	0.208	0.164	0.152	0.146	0.132	0.176
BF1	0.491	1.727	0.494	1.727	0.491	0.604	0.922
BF2	0.354	0.583	0.306	0.583	0.354	0.606	0.464
PM	**0.201**	**0.176**	**0.144**	**0.140**	**0.136**	**0.130**	**0.155**

**Table 3:** OSS separation results: SI-SDR (dB). This accuracy metric indicates the separation performance using the waveforms of the input and predicted audio.

Method	$30^{\circ }$	$60^{\circ }$	$90^{\circ }$	$120^{\circ }$	$150^{\circ }$	$180^{\circ }$	Ave
CM1 [7]	−2.9	−2.5	−2.6	−2.7	−2.8	−3.7	−2.9
CM2 [8]	−2.7	−2.5	−2.5	−2.5	−2.5	−2.5	−2.5
CM3 [11]	4.0	5.3	5.8	5.8	5.7	5.7	5.4
CM4 [13]	**4.8**	5.3	5.5	5.6	5.1	5.6	5.3
AB1	3.0	5.2	5.5	5.5	5.7	5.8	5.1
AB2	3.9	5.1	5.5	5.6	5.6	5.7	5.2
AB3	3.6	4.5	5.4	5.6	5.4	5.7	5.0
MIX	−0.1	−0.5	−1.0	−1.2	−1.5	−1.8	−1.0
BF1	0.1	−20.2	0.1	−20.2	0.1	−1.6	−7.0
BF2	2.5	−1.3	3.6	−1.3	2.5	−1.6	0.7
PM	**4.8**	**5.4**	**6.0**	**6.0**	**6.0**	**6.0**	**5.7**

**Table 4:** OSS separation results: SI-SIR (dB). This accuracy metric indicates the separation performance using the waveforms of the input and predicted audio.

Method	$30^{\circ }$	$60^{\circ }$	$90^{\circ }$	$120^{\circ }$	$150^{\circ }$	$180^{\circ }$	Ave
CM1 [7]	17.6	18.3	16.7	17.2	17.1	16.9	17.3
CM2 [8]	16.8	16.9	16.6	16.6	16.4	16.4	16.6
CM3 [11]	22.1	**31.8**	**34.0**	**33.3**	**32.4**	**32.2**	31.0
CM4 [13]	28.5	32.6	31.4	31.6	27.2	29.1	30.1
AB1	25.8	31.0	31.8	32.1	31.3	28.8	30.1
AB2	22.3	32.0	32.9	32.3	29.9	29.4	29.8
AB3	**28.6**	31.1	29.6	30.9	31.6	30.4	30.4
BF1	1.9	2.6	3.5	2.6	1.9	0.2	2.1
BF2	22.2	24.8	25.8	24.8	22.2	−0.3	19.9
PM	27.9	31.7	33.2	32.8	31.2	30.8	**31.3**

**Table 5:** OSS separation results: SNR (dB). This accuracy metric indicates the separation performance using the waveforms of the input and predicted audio.

Method	$30^{\circ }$	$60^{\circ }$	$90^{\circ }$	$120^{\circ }$	$150^{\circ }$	$180^{\circ }$	Ave
CM1 [7]	−0.1	0.0	0.1	0.1	0.2	0.3	0.1
CM2 [8]	0.3	0.2	0.2	0.2	0.2	0.2	0.2
CM3 [11]	5.4	**6.6**	7.4	7.5	7.6	7.8	7.0
CM4 [13]	5.9	6.5	7.0	7.3	7.1	7.6	6.9
AB1	4.7	6.3	7.0	7.2	7.6	7.9	6.8
AB2	5.3	6.5	7.0	7.4	7.6	7.8	6.9
AB3	4.9	5.8	6.9	7.2	7.4	7.8	6.7
BF1	2.0	−3.5	1.9	−3.5	2.0	1.0	0.0
BF2	3.6	2.0	4.4	2.0	3.6	1.0	2.8
PM	**6.0**	6.5	**7.5**	**7.6**	**7.8**	**8.0**	**7.2**

## Data Availability

Publicly available datasets were analyzed in this study. This data can be found here: https://github.com/facebookresearch/FAIR-Play (accessed on 4 May 2023).
